# Urinary incontinence 12 years after obstetric anal sphincter injury in a longitudinal case control study

**DOI:** 10.1038/s41598-026-36123-y

**Published:** 2026-01-13

**Authors:** Maud de Rham, Baptiste Tarasi, Karine Lepigeon, Chahin Achtari, David Baud

**Affiliations:** Materno-Fetal and Obstetric Research Unit, Woman-Mother-Child Department, University Hospital of Lausanne (CHUV), Lausanne, 1011 Switzerland

**Keywords:** Urinary incontinence, Obstetric anal sphincter injury, Perineal lacerations, Third- and fourth-degree tear, Vaginal delivery, Outcomes research, Urological manifestations

## Abstract

Evidence on long-term urinary incontinence (UI) after obstetric anal sphincter injury (OASIS) is scarce. This longitudinal case-control follow-up study assessed the evolution of urinary symptoms and their impact on quality of life 12 years after vaginal delivery in women with and without OASIS, comparing the findings with previous data from the same study population. A questionnaire, including the Urogenital Distress Inventory (UDI-6; evaluating the degree of bother caused by UI) and the Incontinence Impact Questionnaire (IIQ-7; evaluating the impact of UI on quality of life), was mailed to 242 previous participants (63 women with OASIS and 179 matched controls). The participation rate reached 76%. At 12 years postpartum, mean IIQ-7 scores were similar between groups (1.1 ± 2.6 for OASIS vs. 0.8 ± 1.7 for controls, *p* = 0.300). The UDI-6 scores increased significantly across the entire cohort (2.5 at 6 years vs. 3.3 at 12 years, *p* < 0.001), with a more pronounced rise in the control group (2.3 at 6 years vs. 3.2 at 12 years, *p* < 0.001) compared to the OASIS group, where the difference was not statistically significant (3.0 at 6 years vs. 3.5 at 12 years, *p* = 0.379). At 12 years, the severity of urinary symptoms and their impact on quality of life were comparable between women with OASIS and those without, contrasting with findings at 6 years. Within the entire cohort, the severity of UI symptoms progressed significantly over time.

**Supplementary Information:**

The online version contains supplementary material available at 10.1038/s41598-026-36123-y.

## Introduction

Urinary incontinence (UI) is defined as the involuntary leakage of urine^1^. In women, UI prevalence increases with age, commonly reported around 25–45% in epidemiological studies^2^, and in a national survey of > 5,000 US women, the prevalence was estimated at 61.8%^3^. Its occurrence has a significant negative impact on quality of life^4^. Pregnancy is known to be one of the greatest risk factors for the development of UI. This risk is further elevated in cases of birth weight > 4000 g and when childbirth involves vaginal delivery, instrumental delivery, and perineal tears^5–7^.

In a previous study, we demonstrated that 6 years after the index delivery, women with obstetric anal sphincter injury (OASIS), defined as third- and fourth-degree perineal lacerations, were more likely to experience frequent urination and reported more episodes of urine leakage during physical activity at home compared to women without OASIS^8^. These findings align with the data available in the literature and can be explained by the anatomical and functional interdependence of the pelvic floor organs. However, most of the available studies focus solely on the presence of UI in the short-term following OASIS, leaving a gap in knowledge concerning its long-term progression^9–11^.

OASIS occurs in 0.5% to 4.4% of vaginal deliveries, depending on the region^12,13^. Various risk factors are associated, including nulliparity, advanced maternal age, macrosomia, occiput posterior presentation, prolonged labor, and instrumental vaginal delivery^5,13–16^.

Whether the difference in urinary symptoms between patients with and without OASIS worsens or improves over time remains unknown. This longitudinal case-control follow-up study aimed to assess long-term UI and its progression over time in the same cohort of patients, 12 years after the index delivery.

## Materials and methods

For our initial case-control study^8^, we utilized the database of the obstetrics department of the Centre Hospitalier Universitaire Vaudois (CHUV) in Lausanne, Switzerland. Available data included socio-demographic and pregnancy-related characteristics, as well as perinatal outcomes. All information regarding patient health and their pregnancy was collected upon hospital admission and completed after delivery by the obstetrical care provider.

Women aged 18 or over who gave birth vaginally to a singleton fetus in cephalic presentation between 1996 and 2006 were included, resulting in a total of 13,036 participants. Women under 18 years of age were excluded, as were those with breech presentation or multiple gestation. Within this population, 1.5% (*n* = 196) of the deliveries were complicated by OASIS. These perineal lacerations were all managed using the overlap repair technique, as taught in our hospital at that time^17^.

To assess UI, the identical questionnaire used in our previous study including information on the patient’s current social, demographic, and physical characteristics, as well as the Urogenital Distress Inventory (UDI-6) and the Incontinence Impact Questionnaire (IIQ-7) was mailed to the same 242 participants (63 women with OASIS and 179 matched controls) of our initial case-control study^8^. Of note, matched controls were selected with consideration for age, parity, ethnicity, year of delivery, birth weight, previous caesarean section, mode of vaginal delivery, and episiotomy.

The UDI-6 evaluates the degree of bother caused by UI symptoms, with higher scores indicating a greater degree of incontinence. The IIQ-7 assesses the impact of UI on activities, roles, and emotional states, with higher scores reflecting a greater impact on quality of life. Both questionnaires categorize responses based on the frequency of symptoms or level of discomfort (not at all/slightly/moderately/greatly)^18,19^.

The questionnaire was resent to non-responders after two months, followed by a phone call. Informed consent was obtained from all subjects participated in the study. We estimated a necessary sample size of 47 women with OASIS and 141 controls to achieve an 80% power in detecting a 20% difference with a significance level of 0.05. Anticipating a participation rate of 70%, the sample size expected should meet the objective.

Once the questionnaires were returned, sociodemographic and obstetrical data were compared between the OASIS and matched control groups. Mean scores and score of individual items of the UDI-6 and IIQ-7 were compared between women with and without OASIS. Of note, responses of “moderately” or “greatly” on each item of the UDI-6 and IIQ-7 were considered positive and included in the percentage calculations. The evolution of UI symptoms at 6 and 12 years after the index delivery was investigated.

For statistical analysis, Pearson’s Chi-squared test and Fisher’s exact test were used for categorical variables. Continuous variables were compared using Student’s t-test when normally distributed and the Wilcoxon rank-sum (Mann–Whitney) test when non-normally distributed. Paired comparisons between 6 and 12 years were performed using the Wilcoxon signed-rank test. Statistical analyses were performed using STATA-13 (Stat Corporation, College Station, USA).

The study was carried out in accordance with relevant guidelines and regulations (Declaration of Helsinki) and approved by the local IRB (Ethical Commission of the Canton of Vaud, Switzerland, protocol no. 101/08).

## Results

**Fig. 1:**
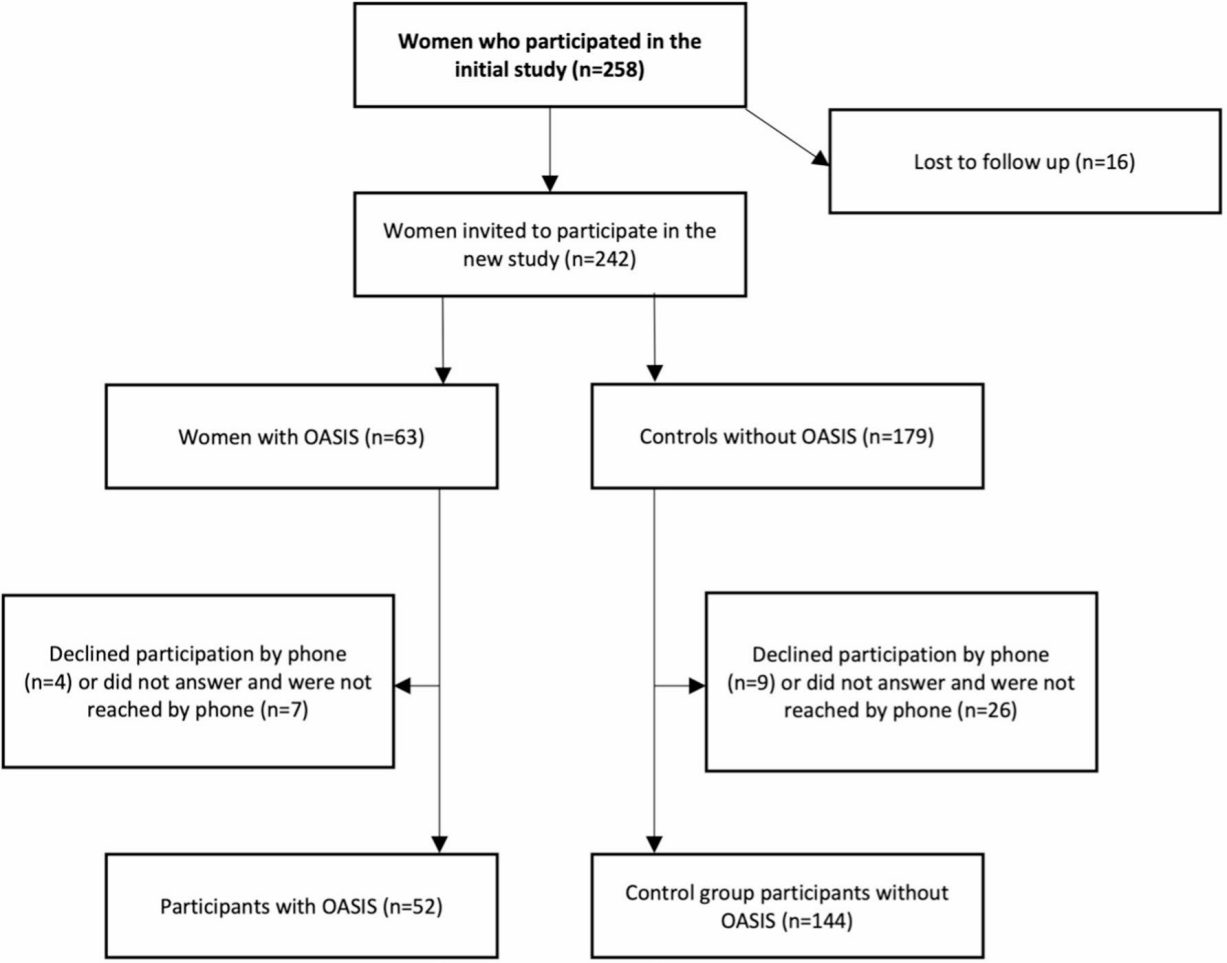
Flowchart of study participant recruitment.

Among the 258 women from our first study^8^, 16 women (6%) could not be located and were classified as “lost to follow up”, 46 women (18%) did not respond after being contacted, and 196 women sent back the completed questionnaire leading to a participation rate of 76%. Specifically, 52 of the 66 women (79%) in the OASIS group and 144 of the 192 women (75%) in the control group returned the questionnaire (Fig. 1).

There were no statistically significant differences in obstetrical data between women with and without OASIS at the time of index delivery (Supplementary Table 1).

The mean time between the index vaginal delivery and the response to the present questionnaire was 12 years (standard deviation, SD 2.6). The mean age of women in both groups was 42 years (SD 5.5). Sociodemographic characteristics at the time women answered the questionnaires were similar between both groups (Supplementary Table 2).

The mean IIQ-7 score was similar in both groups (1.1 ± 2.6 with OASIS versus 0.8 ± 1.7 for controls, *p* = 0.300). Unlike findings from 6 years after delivery, women with OASIS no longer experienced significantly higher frequencies of urinary leakage during physical activities at home (Table 1).

**Table 1:** UDI-6 IIQ-7 results.

Moderate to great symptoms	OASIS (*n* = 52) %	Controls (*n* = 144) %	*p* Value	RR	95% CI
Urinary distress inventory (UDI-6)					
Frequent urination	25.0	22.9	0.761	1.09	0.62–1.91
Urine leakage related to urgency	15.4	13.9	0.792	1.11	0.52–2.36
Urine leakage related to physical activity	21.2	18.8	0.707	1.13	0.60–2.11
Small amounts of urine leakage (drops)	19.2	11.8	0.183	1.63	0.80–3.33
Difficulty emptying bladder	7.7	5.6	0.736	1.38	0.44–4.41
Lower abdominal or genital pain	5.8	7.7	0.764	0.76	0.22–2.60
Mean UDI-6 score (95% CI)	3.5 ± 3.9	3.2 ± 3.1	0.604		
Incontinence impact questionnaire (IIQ-7)					
Urine leakage during:	OASIS (*n* = 52) %	Controls (*n* = 144) %	*p* Value	RR	95% CI
Physical activities at home	3.9	2.8	0.657	1.38	0.26–7.34
Physical activities outside the home	19.2	13.9	0.359	1.38	0.69–2.76
Entertainment activities (cinema…)	5.8	2.1	0.191	2.77	0.58–13.29
Travel longer than 30 min	5.8	2.8	0.385	2.08	0.48–8.97
Social activities	3.9	2.1	0.610	1.85	0.32–10.74
Feeling anxious or depressed	21.2	13.9	0.218	1.52	0.78–2.96
Feeling frustrated	17.3	14.6	0.640	1.19	0.58–2.42
Mean IIQ-7 score (95% CI)	1.1 ± 2.6	0.8 ± 1.7	0.300		

**Fig. 2:**
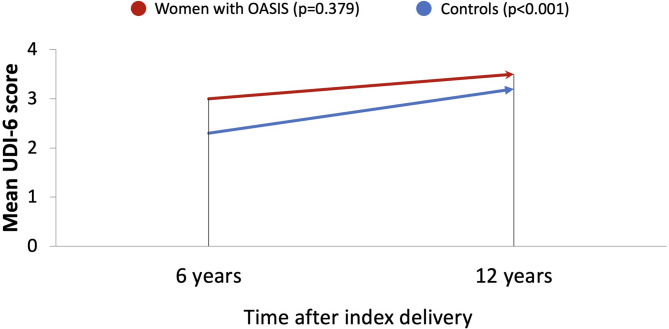
Progression of urinary symptoms.

Regarding the progression of UI symptoms from 6 to 12 years, the mean UDI-6 score increased significantly for the entire cohort (2.5 at 6 years vs. 3.3 at 12 years, *p* < 0.001). Similarly, the mean UDI-6 score in the control group demonstrated a significant increase (2.3 at 6 years vs. 3.2 at 12 years, *p* < 0.001). The OASIS group showed a tendency towards progression in UDI-6 scores, though the increase was less pronounced than in the control group and was not statistically significant (3.0 at 6 years vs. 3.5 at 12 years, *p* = 0.379) (Fig. 2).

OASIS was not associated with an increased prevalence of severe symptoms of urinary incontinence, defined as a UDI score ≥ 4 (25.4% vs. 26.2%, *p* = 0.902) (Supplementary Table 3).

## Discussion

In our population, OASIS occurred in 1.5% of women who gave birth vaginally to a singleton fetus in cephalic presentation. This rate aligns with previously published data^12^.

Our study reveals that at 12 years after childbirth, the severity of UI symptoms and their impact on quality of life do not affect women with OASIS to a greater extent than those without. Unlike after 6 years, women with OASIS were not more likely to experience frequent urination and did not report more frequent urine leakage during physical activity at home.

Our findings are consistent with those of Nilsson et al. and de Leeuw et al., who reported no increase in the long-term prevalence of urinary incontinence following OASIS, supporting the interpretation that such symptoms are more likely to result from other types of pelvic floor injury rather than from obstetric anal sphincter injuries^20,21^. We will mention that levator ani avulsion, often associated with OASIS, contributes to pelvic floor dysfunction and may influence urinary incontinence, although further studies are needed to confirm this association^22^.

Within the entire cohort, severity of UI symptoms progressed significantly over time. Age, which is also known to be a major risk factor for UI^2^, seems to come into play to a greater extent 6 years after our first investigation and 12 years after the index delivery. Chronic damage to the arcus tendinous fasciae pelvis or the paravaginal tissue due to sustained pelvic pressure and strain (e.g., pregnancy, chronic coughing associated with pulmonary diseases, chronic constipation, and other age-related conditions), may account for the increased severity of UI symptoms^23^.

We will also mention that BMI tends to increase with age, and higher BMI has been associated with a greater prevalence of urinary incontinence^24^. Although not statistically significant, the control group included a higher proportion of women with a BMI > 30 and was therefore considered as a potential confounder. However, after adjustment for BMI, the effect estimates (adjusted relative risks) were materially unchanged compared with the unadjusted analysis. This indicates that BMI did not confound the association between OASI and urinary incontinence outcomes in our cohort.

The prevalence of UI among postpartum women is high. Since this condition significantly impacts quality of life^4^, these findings highlight the importance of implementing effective preventive measures. Strengthening the pelvic floor muscles early in pregnancy has been shown to reduce the rate of postpartum UI^25^.

For the conservative management of UI, intensive and supervised pelvic floor muscle strengthening has also demonstrated significant effectiveness. It is important to highlight that women with OASIS were significantly more likely to have received physiotherapy compared to those in the control group, which may have contributed to the observed results. Other interventions, such as electrical stimulation or the use of cones, have also proven beneficial. Lifestyle modifications such as weight loss are impactful too^26^.

UI is likely underestimated because patients often avoid discussing symptoms they find embarrassing^27^. Gynecologists and general physicians should proactively investigate the presence of this condition.

The ability to analyze the long-term evolution of our cohort 12 years after the index delivery is the main strength of this study, adding to limited long-term data within the literature regarding OASIS and UI. The high response rate, reaching 76% is another strength. Regarding the limitations, we will point out the moderate number of participants. The absence of statistical significance for individual IIQ-7 items may be related to limited statistical power, as the study was designed to detect differences in the total IIQ-7 score, while analyses of individual items were secondary. Moreover, as OASIS may be underdiagnosed^28^, their prevalence is therefore likely to be underestimated^29^, resulting in an inevitable classification bias that might reduce the accurate assessment of the impact of these injuries. In addition, baseline symptoms were not evaluated through our questionnaires, making it difficult to determine whether these symptoms were present before the index delivery. We were also unable to determine whether patients experienced subsequent third- or fourth-degree perineal tears, as delivery records for later births, some occurring in other hospitals, were not available. Finally, pelvic organ prolapse was not evaluated, which represents a limitation given its close relationship with pelvic floor function and its possible influence on urinary symptoms.

## Conclusion

Twelve years after vaginal delivery, the severity of UI symptoms and their impact on quality of life were similar between women with and without OASIS, in contrast to findings at 6 years, when those with OASIS were more affected. Throughout the cohort, UI symptoms worsened significantly over time, indicating that aging has a major influence on this condition.

## Supplementary Information

Below is the link to the electronic supplementary material.

Supplementary Material 1

## Data Availability

The data sets was used and analyzed during the current study are available from the corresponding author upon reasonable request.

## Abbreviations

UI: Urinary incontinence
OASIS: Obstetric anal sphincter injury
