# Supplementary material for: Urinary incontinence 12 years after obstetric anal sphincter injury in a longitudinal case control study
Source: Sci Rep. 2026 Jan 13;16:5179. doi: 10.1038/s41598-026-36123-y (PMC12881629; doi:10.1038/s41598-026-36123-y)
Supplement: Supplementary file 1 — Supplementary Material 1 [file 41598_2026_36123_MOESM1_ESM.docx]

**Supplementary Table 1. Obstetrical data at the index delivery**

|  | **OASIS** | **Controls** | **p-value** |
| --- | --- | --- | --- |
|  | **(n=52) %** | **(n=144) %** |  |
| Previous Cesarean Section | 3.9 | 5.6 | 1.000* |
| Induction of labor | 34.6 | 32.2 | 0.748 |
| Epidural analgesia | 73.1 | 69.9 | 0.669 |
| Prolonged second stage >2 h | 25 | 31.5 | 0.382 |
| Mediolateral episiotomy | 38.5 | 49 | 0.194 |
| Persistent occiput posterior position | 19.2 | 12.6 | 0.429 |
| Type of vaginal delivery |  |  |  |
| Normal | 67.3 | 59.7 | 0.669* |
| Forceps | 30.8 | 38.2 |  |
| Vacuum | 1.9 | 2.1 |  |
| Birth weight >90th percentile | 9.6 | 11.1 | 0.765 |
|  | **p-value using Fisher's exact* | | |

**Supplementary Table 2. Sociodemographic characteristics**

|  | **OASIS** | **Controls** | **p-value** |
| --- | --- | --- | --- |
|  | **(n=52) %** | **(n=144) %** |  |
| Age (years+SD) | 42 ± 5.7 | 41.9 ± 5.4 | 0.906 |
| Nationality |  |  |  |
| Swiss | 69.2 | 56.3 | 0.215 |
| Non-Swiss | 30.8 | 43.7 |  |
| Parity at the time of the questionnaire | 2.1 ± 0.8 | 2.1 ± 0.68 | 0.895 |
| 1 | 19.2 | 14.6 | 0.431 |
| ≥ 2 | 80.8 | 85.4 |  |
| Body mass index ≥ 30 | 2 | 9.8 | 0.122 |
| Menopause | 7.8 | 4.3 | 0.461* |
| Gynecological surgery after index birth | 7.8 | 16.4 | 0.163* |
| Physiotherapy | 59.6 | 25.2 | 0,001 |
| Taking oral contraception | 7.7 | 7.6 | 1.000* |
| ≥ 1 Cesarean section | 28.9 | 17.4 | 0.078 |
| Smokers | 13.5 | 11.9 | 0.767 |
| University degree | 21.2 | 11.2 | 0.075 |
| Martial status (married) | 86.5 | 84.6 | 0.739 |
| Work status (employed) | 95.9 | 90.7 | 0.361 |
| Health insurance |  |  |  |
| Non-private | 96.2 | 95.8 | 1.000* |
| Private | 3.8 | 4.2 |  |
|  | **p-value using Fisher's exact* | | |

**Supplementary Table 3. Severe Urinary Incontinence in the whole cohort (defined as UDI score ≥4)**

|  | **UDI score < 4** | **UDI score ≥ 4** | **p-value** |
| --- | --- | --- | --- |
|  | **(n = 129) %** | **(n = 67) %** |  |
|  |  |  |  |
|  |  |  |  |
| OASIS | 26,2% | 25,4% | 0,902 |
|  |  |  |  |
| Age ≥ 40 (years) | 22,2% | 31,3% | 0,166 |
|  |  |  |  |
| Nationality |  |  | 0,937 |
| Swiss | 69,6% | 70,2% |  |
| Non-Swiss | 30,4% | 29,9% |  |
|  |  |  |  |
| Parity at the time of the questionnaire |  |  | 0,328 |
| 1 | 12,7% | 17,9% |  |
| >2 | 87,3% | 82,1% |  |
|  |  |  |  |
| Body mass index >30 | 7,3% | 9% | 0,677 |
|  |  |  |  |
| Smokers | 5,6% | 22,4% | 0,001 |
|  |  |  |  |
| University degree | 16% | 10,5% | 0,291 |
|  |  |  |  |
| Marital status |  |  | 0,394 |
| Unmarried | 12% | 16,4% |  |
| Married | 88% | 83,6% |  |
|  |  |  |  |
| Work status - Unemployed | 5,6% | 10,5% | 0,218 |
|  |  |  |  |
| Health insurance (private) | 4% | 4,5% | 1,000 |
|  |  |  |  |
| Menopause | 4,9% | 6,1% | 0,742 |
|  |  |  |  |
| Gynecological surgery after index delivery | 11,4% | 20% | 0,109 |
|  |  |  |  |
| Physiotherapy | 32,5% | 38,5% | 0,415 |
|  |  |  |  |
| Taking contraception | 7,1% | 9% | 0,654 |
|  |  |  |  |
| Previous C Section | 20,6% | 20,9% | 0,966 |
|  |  |  |  |
| Induction of labor | 56,8% | 61,2% | 0,556 |
|  |  |  |  |
| Epidural analgesia | 72% | 70,2% | 0,787 |
|  |  |  |  |
| Prolonged second stage >2 h | 28,8% | 32,8% | 0,562 |
|  |  |  |  |
| Mediolateral episiotomy | 40,8% | 56,7% | 0,035 |
|  |  |  |  |
| Persistent occiput posterior position | 12% | 17,9% | 0,416 |
|  |  |  |  |
| Instrumental delivery | 40,3% | 43,8% | 0,655 |
|  |  |  |  |
| Birth weight >90th percentile | 11,1% | 10,5% | 0,888 |
